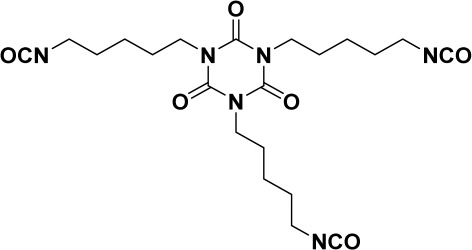# State-of-the-Art Polyurea Coatings: Synthesis Aspects, Structure–Properties Relationship, and Nanocomposites for Ballistic Protection Applications

**DOI:** 10.3390/polym16040454

**Published:** 2024-02-06

**Authors:** Gabriela Toader, Aurel Diacon, Sorin Mircea Axinte, Alexandra Mocanu, Edina Rusen

**Affiliations:** 1 Military Technical Academy “Ferdinand I”, 39-49 George Coșbuc Boulevard, 050141 Bucharest, Romania; nitagabriela.t@gmail.com (G.T.); aurel_diacon@yahoo.com (A.D.); 2 Faculty of Chemical Engineering and Biotechnologies, University Politehnica Bucharest, Gh. Polizu Street, 011061 Bucharest, Romania; alexandra.mocanu@imt.ro; 3 S.C. Daily Sourcing & Research SRL, 95-97 Calea Griviței, 010705 Bucharest, Romania; sorinaxinte@yahoo.com; 4 National Institute for Research and Development in Microtechnologies—IMT Bucharest, 126A Erou Iancu Nicolae Street, 077190 Bucharest, Romania

**Keywords:** polyurea, composites, ballistic protections, step-growth polymerization

## Abstract

This review presents polyurea (PU) synthesis, the structure–properties relationship, and characterization aspects for ballistic protection applications. The synthesis of polyurea entails step-growth polymerization through the reaction of an isocyanate monomer/prepolymer and a polyamine, each component possessing a functionality of at least two. A wide range of excellent properties such as durability and high resistance against atmospheric, chemical, and biological factors has made this polymer an outstanding option for ballistic applications. Polyureas are an extraordinary case because they contain both rigid segments, which are due to the diisocyanates used and the hydrogen points formed, and a flexible zone, which is due to the chemical structure of the polyamines. These characteristics motivate their application in ballistic protection systems. Polyurea-based coatings have also demonstrated their abilities as candidates for impulsive loading applications, affording a better response of the nanocomposite-coated metal sheet at the action of a shock wave or at the impact of a projectile, by suffering lower deformations than neat metallic plates.

## 1. Introduction

**Scheme 1:**
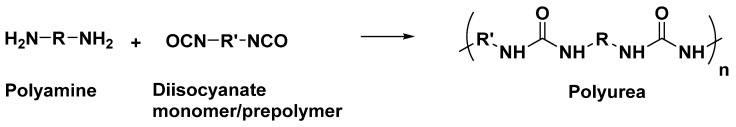
General synthesis scheme of polyurea by polyaddition of diisocyanates and diamines.

Polyurea (PU) polymers were first mentioned in the literature in 1948, when authors compared the thermal properties of different polymers/fibers [1,2]. However, despite the patent application of Bayer in the 1950s [3,4], polyurea received real, intense commercial attention in the late 1980s, following the efforts made by Barton and Schlichter [5] and Texaco Chemical Company [6], which aimed to develop polyurea as a complementary use of their patented raw material known as Jeffamine^®^. Polyurea synthesis entails step-growth polymerization through the reaction of an isocyanate monomer/prepolymer and a polyamine, each one possessing a functionality of at least 2, in accordance with the reaction presented in Scheme 1. The advantage of this reaction is that it does not require heat or a catalyst [7].

Commercial polyurea formulations differ according to the amine resin composition, which consists of a long-chain diamine (soft segment), a short-chain diamine (chain extender), and the diisocyanate component [8]. One of the key ingredients in the production of polyurea is represented by isocyanates, which can be categorized as aliphatic, aromatic, bifunctional, or heterobifunctional by nature. Toluene diisocyanate (TDI), 4,4’-diphenylene methane diisocyanate (MDI), naphthalene diisocyanate (NDI), and p-phenylene diisocyanate (pPDI) are examples of aromatic isocyanates used for obtaining polyurea coatings. The most commonly used aliphatic isocyanates are 1,6-hexane diisocyanate (HDI) and isophorone diisocyanate (IPDI). High-molecular-weight amine-terminated polyethers are employed in polyurea synthesis, but also low-molecular-weight amines, e.g., diethylenetriamine (DETA) and triethylenetetramine (TETA), are frequently utilized as chain extenders for aliphatic polyurea, while diethyl-toluenediamine (DETDA) or dimethylthio-toluenediamine (DMTDA) is included in the formulations for aromatic polyurea. Aromatic amines and the compounds that possess secondary amine functionalities slow down the polymerization reaction remarkably [8], allowing the processability of the reaction mixture and an easier application of the coating on the targeted substrate.

Polyureas are amongst the most advantageous materials in the coatings industry and are used in a variety of sectors due to their exceptional adherence to a wide range of surfaces, fast drying time, chemical resistance, and low flammability. The applications of polyurea include use as materials for wood, concrete, and steel coating with anticorrosive and scratch-resistant properties. This type of polymer can be utilized both for indoor and outdoor products resistant to weathering and UV degradation and displays adhesive properties that facilitate its application. Furthermore, polyurea display good resistance properties to different chemical reagents, oils, etc. [9]. Polyurea has a complex mechanical reaction under both static and dynamic large-strain loading situations, which can be attributed to its hierarchical microstructure [10].

Pure polyurea coatings constitute optimal insulation layers, making polyurea linings one of the best options for flooring in buildings, with numerous benefits [8]. Due to their many uses, including in parking lots, halls, swimming pools, factories, and coatings for sport areas (such as outdoor playgrounds with shock absorbers), polyurea linings are highly valued for their exceptional mechanical properties, wear resistance, and lack of sensitivity to moisture during the curing process. The polyurea coating application processes fall into two categories: hot polyurea and cold polyurea. The pure form of polyurea is called hot polyurea, while cold polyurea, sometimes referred to as hybrid polyurea, is a mixture of polyurethane and polyurea. Hot polyurea is utilized for specialized and industrial uses (such as construction applications), while cold polyurea is typically used for domestic applications. The utilization of complex equipment is not necessary when applying cold polyurea. The application of the coating can be carried out with standard equipment like rollers. However, pure polyurea must be applied using certain tools that provide heat and allow spraying. Many applications, such as corrosion protection, lining, membranes, and sealants, use this technique because of the unique curing profile and exceptional qualities of the polymeric film [8].

Some of the general characteristics of typical polyurea are presented in Table 1 [8].

**Figure 1:**
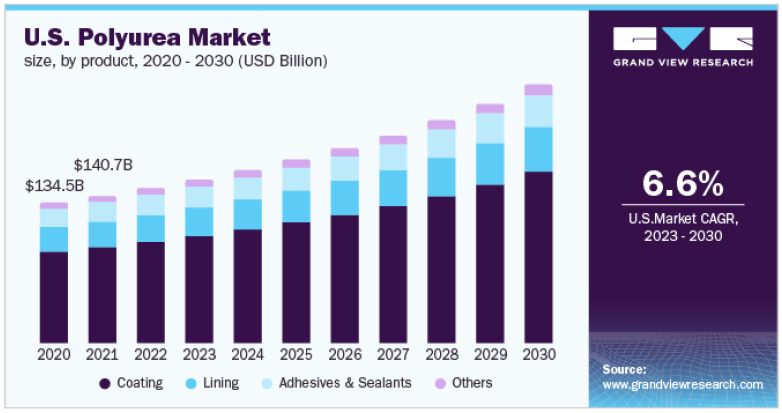
The US polyurea market size and predictions (adapted from [11]).

The US polyurea market is experiencing continuous development; the evolution for 2020–2023 and the prediction up to 2030 are presented in Figure 1 [11]. 

## 2. Polyurea Coatings for Ballistic Protection Applications

### 2.1. Overview

One of the most important applications of polyurea coatings is represented by ballistic protection [12,13,14]. This is motivated by the existence of the present conflict areas. Ballistic protection is frequently necessary for troops, police officers, and general security personnel. Ballistic protection includes apparel, vests, armors, and helmets, but also structural reinforcement for vehicles [15].

The development of the formulation of a composition for the polyurea copolymer and the mechanisms that arise during the detonation, deflagration, and impact moment should be understood. 

Nonlinear, high-amplitude, extremely short-lived acoustic pressure waves are known as shock waves. Debilitating health effects arise from blast-induced traumatic injury caused by exposure to high-energy explosives that generate shock waves [16]. Innovative methods for shock wave energy dissipation (SWED) must be developed because conventional impact-absorbing materials fail to attenuate shock waves adequately. Commercial polymers with microphase-segregated hard and soft domains, such as polyurea, represent the state-of-the-art materials for SWED nowadays. Impact-induced glass transitions, dynamic hydrogen bonding, and wave scattering at hard/soft interfaces have all been associated with the exceptional dissipative performances of polyurea matrix [17,18,19]. Similar to the hard/soft domains of polyurea, semicrystalline materials like the ultra-high-molecular-weight polyethylene (UHMwPE) fiber branded as Dyneema^®^ have amorphous/crystalline boundaries that can dissipate shock waves [20].

**Scheme 2:**
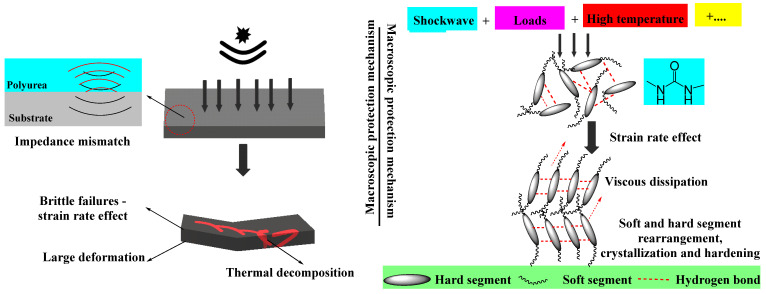
Schematic illustration of the shock wave mitigation effect of polyurea coatings (adapted from [2]).

Relatively recent studies [21,22] sustain that the explosion shock wave determines a reordering at a molecular level; thus, the molecular rearrangement and neutralization appear to be events dominated by energy dissipation during impact. This arrangement process is associated with a viscoelastic dissipation of the deformation rate [2], leading to improved mechanical properties and diminished effects of the stress rate and impedance mismatch between support and polymer coating [23] (Scheme 2).

**Scheme 3:**
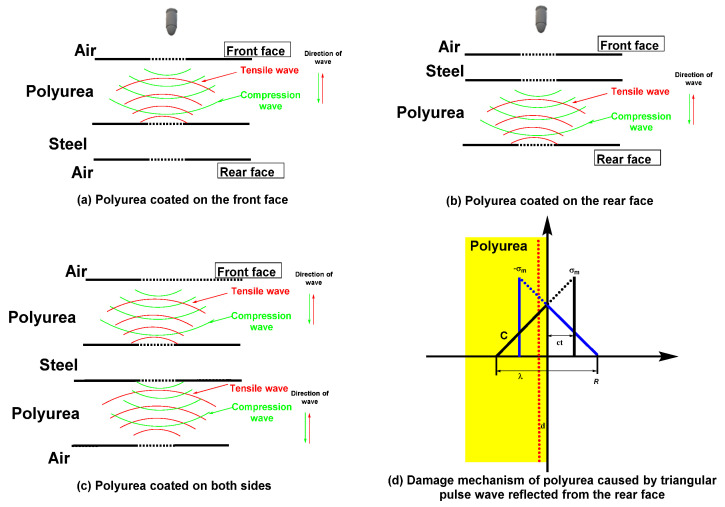
Schematic illustration of anti-penetration performances of polyurea/ASTM1045–steel composite plates (adapted from [28]).

A significant amount of research was conducted on the energy dissipation mechanism of polyurea coatings under the blast overpressure effect. However, there is a relatively small number of published papers that explore the behavior of polyurea-coated metallic substrates subjected to localized loads generated from bullet impact. When subjected to high-velocity impact, polyurea coatings could improve the penetration/fragmentation resistance of the underlying substrate, depending on the following parameters: thickness of the polyurea coating [24], number of layers and configuration of the composite, dissipation of the localized shock wave (Scheme 3) through multiple reflections as it traverses the composite [25], T_g_ and a viscoelastic transition of the elastomer to its glassy state due to the localized loading resulting from the impact of a high-speed projectile [23], dynamics of hard/soft segments and hydrogen bonding [26,27], etc.

Youssef [29] tested sandwich structures comprised of 1–2 mm thick polyurea layers as an inner layer and glass, acrylic, polyurethane, Al, steel, and PMMA panels as outer layers to evaluate the influence of the substrate on the polyurea response to dynamic loadings. The transmitted stress wave amplitude was greatly decreased in both metallic polyurea sandwich structures due to a considerable impedance mismatch between polyurea and metal substrate, which effectively confined the stress wave within the incident substrate. In contrast, a resonance phenomenon was observed when the polyurea layer was placed between the acrylic or polycarbonate plates [29]. Two mechanisms have been reported to explain the stiffening effect induced by the presence of a polyurea as an elastomeric coating for steel plates subjected to impact: (i) its high energy absorption capacity due to resonance between the polymer segmental dynamics and the impact frequency (approx. 10^−5^ s) and (ii) lateral spreading of the force applied due to transient hardening of the coating due to its transition from rubbery to glassy state [30].

### 2.2. Structure–Properties Relationships

The initial microstructure of polyurea, as well as microstructural changes during deformation, has a significant influence on its mechanical properties [31]. 

**Scheme 4:**
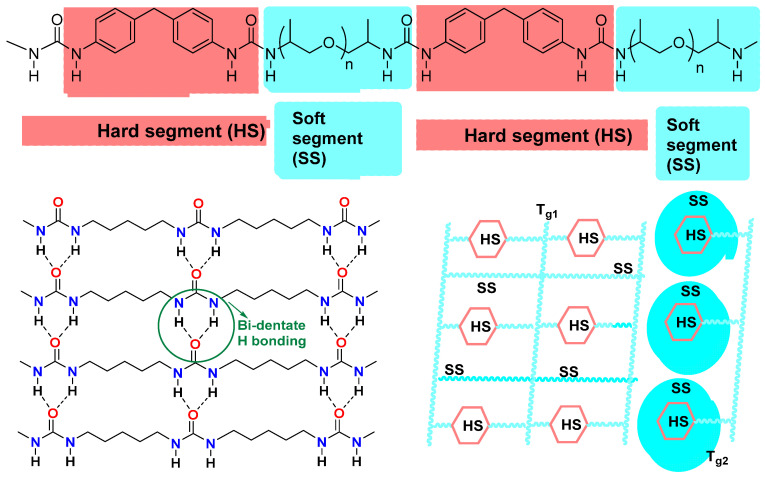
Schematic representation of hydrogen bond formation in polyurea and hard/soft segments, respectively.

By careful selection of the components, particularly by adding chain extenders to the amine co-reactant side of the formulation, the mechanical properties of polyurea can be adjusted. The establishment of urea linkages next to one another is promoted by chain extenders, which leads to the development of H bonds in the hard segments. Even though both aliphatic and aromatic chain extenders are frequently utilized, it seems that the ideal ratio of the two drives the developing macromolecule to orient in a way that promotes the best possible H bonding. The high polarity of the oxygen atoms, and the hydrogen in the urea functional group, determines the formation of hydrogen bonds between the polymer chains, thus explaining why the polyurea copolymers display two T_g_ domains, in correlation with the microphase segregated structures they contain. The lower T_g_ value is usually specific to the polyamine block (soft segment), while the higher value corresponds to the isocyanate block (hard segment) that is incorporated in the first (Scheme 4) [32,33,34]. The chemical incompatibility between the hard and soft segments is generally acknowledged to be the cause of microphase-separated structure formation [35,36,37]. If the segments are just mixed, this incompatibility could lead to macrophase separation. Copolymerization, on the other hand, results in microphase-separated morphology by introducing covalent linking between segments that avoids macroscopic phase separation. The hard domain is often formed by the hard segments self-assembling. However, microphase separation is typically not fully achieved, and some hard segments may be trapped or scattered within the domains of soft segments [38]. A symmetric structure of the diisocyanate can facilitate the self-assembly of the hard domains [39]. The influence of the diisocyanate type on the properties of polyurea is presented in Table 2 [40]. For the presented data (Table 2), it is evident that polyurea based on symmetric diisocyanates have much greater tensile strengths in comparison to those based on unsymmetric diisocyanates. Thus, compared to its homologs based on PPDI, HDI, and CHDI, polyurea based on MPDI, IPDI, and TDI demonstrated much lower tensile strengths. Also, polyurea based on CHDI showed the maximum tensile strength value of 30 MPa.

Hydrogen bonding is critical in the generation of hard domains and microphase segregated structures. Monodentate hydrogen bonding typically characterizes a disordered phase separation. The cohesive strength of the bidentate hydrogen bonding interaction in urea is substantially stronger than the monodentate interaction, favoring mechanical straightening in polyurea [41]. The degree of the tightly ordered hydrogen bond network inside the hard domains and the long-range connectivity of the hard domains can be correlated with stiffness, strength, and high energy dissipation during large deformation [42]. However, a high hardness is not desirable since the slow hard segment dynamics during large deformation (resistance to blast testing) will cause material failure by not efficiently dissipating the stress [43].

**Scheme 5:**
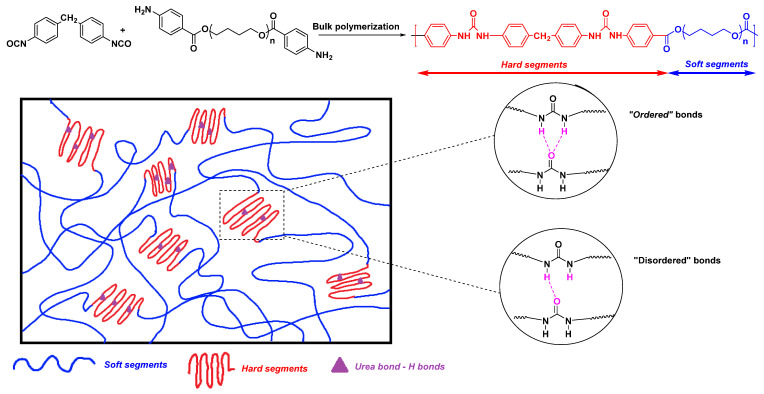
Schematic representation of “ordered” and “disordered” hydrogen bonds (adapted from [42]).

The hard segment dynamics significantly slow down due to the hydrogen bonding, which causes the urea groups to be arranged head to tail (Scheme 5) [42,44]. Therefore, the two glass transition temperatures of polyurea and, consequently, the hydrogen bonding pattern are closely correlated with the existence of the microphase-segregated structure, which is composed of hard nanodomains enclosed in a soft (elastically compliant) matrix [2,45,46,47]. The existence of one or two glass transition temperatures in polyurea matrices is attributed to the degree of segregation of nanodomains, which depends on the volume fraction of hard segments [45] and, subsequently, on the length of the aliphatic chains forming the soft regions, and therefore also on the average distance between the hard nanodomains, which decreases for shorter aliphatic chains. In general, the lowest glass transition is assigned to the aliphatic chains which possess a higher mobility because they are not in the proximity of the hard nanodomains, and the second glass transition temperature corresponds to the chains that have a restricted mobility due to their closeness to hard segments. The stiffening and crosslinking attributes of the hard domains perform as impediments that restrict the mobility of the soft segments in the vicinity of the interface, changing their dynamics [27,48]. To guarantee that the material offers great strength while being able to withstand significant deformation, prevent structural damage, and lower the fragmentation rate, the ratio of hard to soft segments in polyurea should be rationally adjusted [2]. The stiffness of the polyurea changes as the hard domain content increases [49]. Chen et al. [50] studied the two-phase morphology of polyurea within the range 20–80 wt. % of hard segment concentration and showed that, at hard segment concentrations higher than 50–60 wt. %, due to the higher degree of phase mixing in high hard segment concentrations, the polyurea matrix tends to become brittle. The stiffness of the hard domain can be attributed to hydrogen bonds between the C–O groups in urea and ether and the N–H group in urea, as well as potential π–π interactions between adjacent aromatic moieties [51]. The amount of ether–oxygen-bonded N–H diminishes as the hard segment content increases, which implies that the amount of disordered N–H reduces and the amount of ordered N–H grows [52]. To enhance microphase separation and decrease the formation of hydrogen bonds between soft and hard segments, hard segment aggregation is primarily responsible for the ratio shift in ether–oxygen-bonded N–H. When comparing their effects, ordered hydrogen bonding arrangements tend to improve the mechanical characteristics more than disordered hydrogen bonding stacks, which tend to improve self-repairing properties more. Although ordered hydrogen bonds provide materials’ strength, disordered hydrogen bonds have low bond energy and dissipate energy as sacrificial bonds [52].

**Figure 2:**
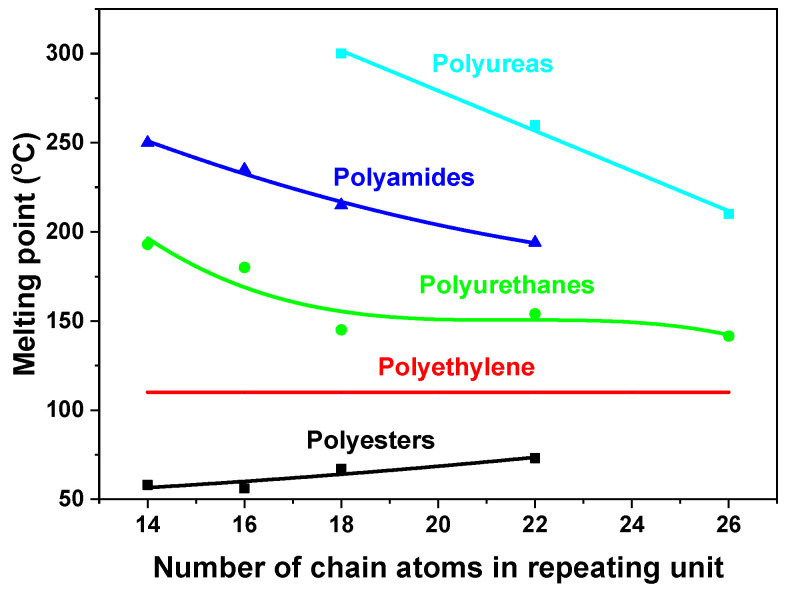
Effect of the number of carbons and hydrogen bonding on the melting point of different classes of polymers (adapted from [1]).

Figure 2 depicts the melting point dependence on the number of carbon atoms in the repeating unit of various types of polymers capable of forming hydrogen bonds [1]. Polyurea present a higher melting point that can be explained by the hydrogen intermolecular bonding between the polymer chains. 

**Figure 3:**
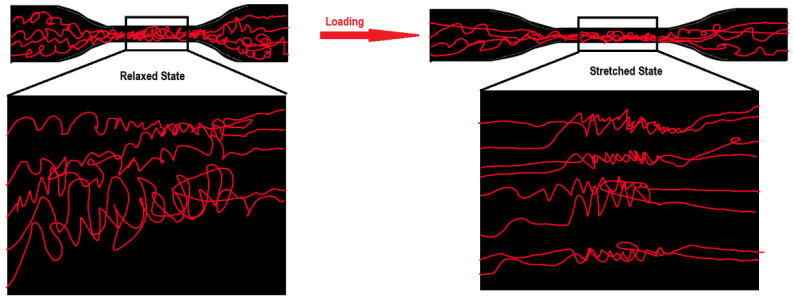
Microstructural changes in polyurea upon tensile loading (adapted from [32]).

The detonation of an explosive charge causes an overpressure that consists of a shock wave and a blast wind [53]. The ballistic protection efficiency is mostly determined by the soft segment qualities rather than hard segment characteristics [54]. In this context, the contribution of the hydrogen bonds is almost negligible. The mechanism most widely acknowledged that explains the ballistic efficiency is the “rubber-to-glass second-order transition” that takes place in the structure of the polyurea at high deformation rates [55]. In these conditions, the rubbery polyamine tends to have a glassy behavior, and the polyurea will suffer fragile cracks. The reorientation of the amorphous segments is not possible in the presence of the load, and a “freeze out” of the chains takes place (Figure 3). These deformation rates lead to a process comparable to a high-T_g_ (but lower than the testing temperature) polymer segment dispersion, which induces the transition from rubber to glassy state, dissipating a significant amount of energy [56]. The hard domains act as a panel (stiffening) as well as the crosslinking between the soft segments, and, in consequence, the mobility of the polyurea molecule is constrained at the interface, thus modifying the dynamic behavior of the material [27,48]. Obviously, the rubbery-to-glassy-state transition influences the adhesion to the support on which the polyurea is deposited [57].

The ratio between the soft and hard segments is extremely important for the ballistic shielding capabilities of the material.

(1) $$Hard\;Segment\;\left(\%\right)=\frac{m_{iso}+m_{ext}}{m_{iso}+m_{ext}+m_{amine}}\times 100$$

where *m_iso_*, *m_ext_*, and *m_amine_* refer to the amount (g) of isocyanate, extender, and amine, respectively.

The chain extender is usually a molecule with a low molecular weight that has the role of increasing the molecular weight of the polyurea by establishing strong bonds between the two types of segments [58]. In addition, it forces the urea bonds closer together and thus facilitates the formation of hydrogen bonds between the hard segments [21,59,60]. 

### 2.3. Specific Properties of Polyurea Coatings Employed for Ballistic Protection Applications

The requirements for polyurea films employed in ballistic protection are as follows: [2]:

- The ratio between the hard and soft segments must be optimized to guarantee a high-strength material that can withstand high deformation and impede structural deterioration;
- They must present high thermal resistance while maintaining their mechanical properties;
- They must display a high elasticity modulus and long plastic stage when subjected to high-strain-rate loading and also a high loss modulus and storage modulus, thus dispersing all the energy during the deformation process.

The main brands of polyurea marketed for ballistic protection and their mechanical properties are presented in Table 3.

#### 2.3.1. Mechanical Properties of Polyurea-Based Films Designed for Ballistic Protection Applications—Tensile Tests

**Figure 4:**
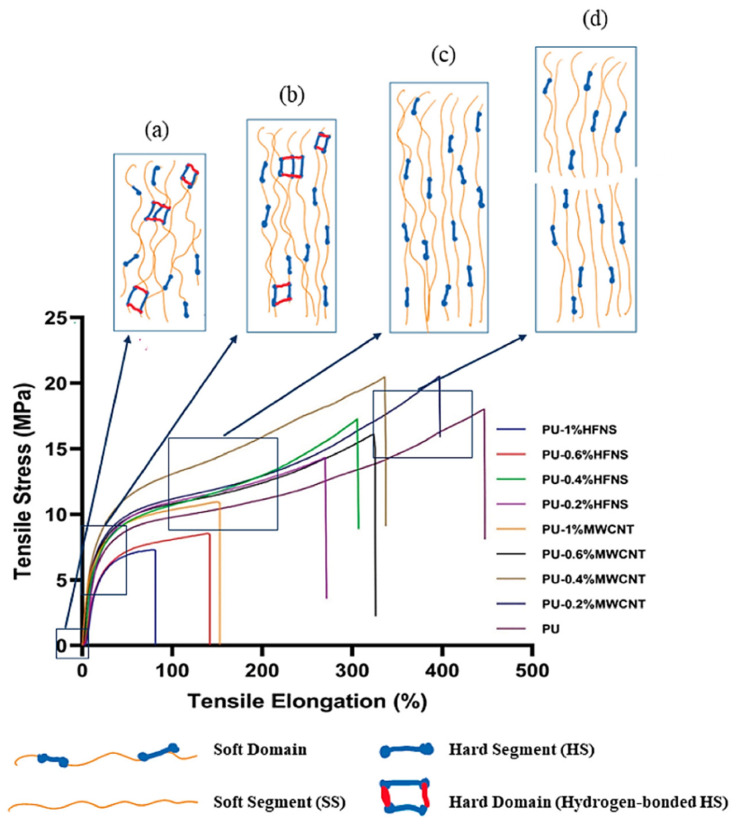
Exemplification of polyurea matrix response during tensile testing: (**a**) chain arrangements at 0%, (**b**) chain motions during elastic deformation, (**c**) breakage of hard domain during plastic deformation, and (**d**) fracture of polyurea chains (from [72]).

Predictive design of PU materials, particularly with a focus on structure, becomes difficult due to the likelihood of both high-pressure and high-strain-rate effects in response to an explosion or bullet impact [70]. According to Leite et al. [71], ideally, the maximum elongation should be as high as possible to allow for deformation and energy dissipation (Figure 4). The stress–strain (elongation) curve presents two domains, an elastic region and a plastic zone. In the elastic zone, the application of stress leads to continuous deformation with recovery when the stress is removed. In the plastic zone, the recovery does not happen when the stress is removed. After entering the plastic stage, the continuous application of the stress leads to the material breaks [72].

The uniaxial strain was observed to considerably disrupt the phase-separated microstructure and chain orientation, resulting in a significant slowing and broadening of the polyurea soft phase segmental relaxation. With increased strain in uniaxial tensile loading, intersegment microphase separation is greatly diminished, increasing the intermixing contribution to the scattering. Hard segments become more closely linked to the soft phase when straining polyurea to significant values due to disruptions in the hard domain orientation [73]. A common technique for investigating hard domain orientation and any alterations in unlike segment mixing is small-angle X-ray scattering (SAXS). The 2D SAXS results show that, in uniaxial tensile deformation, the hard domains, after relaxation, maintain a significant orientation following their displacement by the applied strain [73]. Under compressive loading (hydrostatic compression), the SAXS peak’s loss indicates that polyurea rapidly changes from an ordered, phase-separated state to a disordered, intermixed one, as demonstrated by Rosenbloom et al. [70]. Regardless of the deformation type applied, surpassing the yield stress results in unrecoverable strain and domain orientation. Additionally, the disruption of the hard domains leads to a decrease in the degree of phase segregation [73]. Under compression, the mean spacing of the interchain hydrogen-bonded network within the hard segment domains reduces; in certain situations, it recovers when the load is removed [70]. Additionally, the length of the soft segment determines the degree of microphase separation, which, in turn, determines how much the polyurea deforms and rebounds. Polyurea with a longer soft segment deforms less and recovers more [70].

#### 2.3.2. Viscoelastic Properties of Polyurea-Based Films Designed for Ballistic Protection Applications—Dynamic Mechanical Analysis

DMA experiments can be used to better understand the mechanical response of a polymer under dynamic loadings [74]. Using DMA analysis, the storage modulus can be determined ($E_{p}^{\prime }$) (which describes the elastic behavior of the polyurea), along with the loss modulus ($E_{p}^{\prime \prime }$) (attributed to the energy dissipation capacity of the material) [75]. Thus, the complex Young’s modulus *E_p_* can be expressed as:

(2) $$E_{p}=E_{p}^{\prime }+iE_{p}^{\prime \prime }=E_{p}^{\prime }\left(1+i\eta_{p}\right)$$

(3) $$\eta_{p}=\frac{E_{p}^{\prime \prime }}{E_{p}^{\prime }}$$

where *η_p_* is the damping loss factor of polyurea.

The DMA testing results of polyurea sustain the increase in the storage modulus with the frequency, but there is a decrease with the temperature augmentation, as presented by Wang et al. [75]. The damping loss factor, at a set frequency, increases with the temperature up to a maximum, after which it decreases. This maximum value represents the T_g_ value [76]. It was determined that the soft segment length is directly correlated with the frequency required to start the dynamic “rubber-to-glass” transition process. The loss and storage modulus traces visibly shift toward higher temperature as the frequency of the dynamic loading increases. It should be noted that increasing the testing temperature has the same effect on mechanical characteristics as lowering the testing speed, i.e., the frequency associated with dynamic tests. This unique relationship enables the evaluation of mechanical properties over a broad frequency range by conducting tests at very narrow frequencies. In general, the goal of DMA investigations of polyurea with varying soft segment lengths is to determine the frequency required for the material to go through the dynamic glass transition process. Even when exposed to blast loading conditions, polyurea typically stays in the rubbery regime; as a result, the coating serves as a “catcher system” for the resulting fragments. Concurrently, shock-wave-induced hard domain ordering and crystallization, H bond reorganizations, and shock wave neutralization and/or capture all enhance the capacity of polyurea to withstand blasts. 

Based on the literature, the high modulus of elasticity of polyurea is responsible for the self-sealing characteristic which causes the coating to close on itself in the event of penetration damage [77,78]. Self-healing processes known as intrinsic mechanisms occur inherently and without the involvement of a catalyst or healing agent. These mechanisms rely on the reversible nature of the chemical bonds located in the polymeric matrix. Physical interaction and chemical interaction are two other categories of intrinsic processes. Molecular interdiffusion occurs when molecules interact physically. Dynamic bonding results from chemical interaction [79].

#### 2.3.3. Thermal Properties of Polyurea-Based Films Designed for Ballistic Protection Applications—Differential Scanning Calorimetry (DSC) and Thermogravimetric Analysis (TGA)

**Figure 5:**
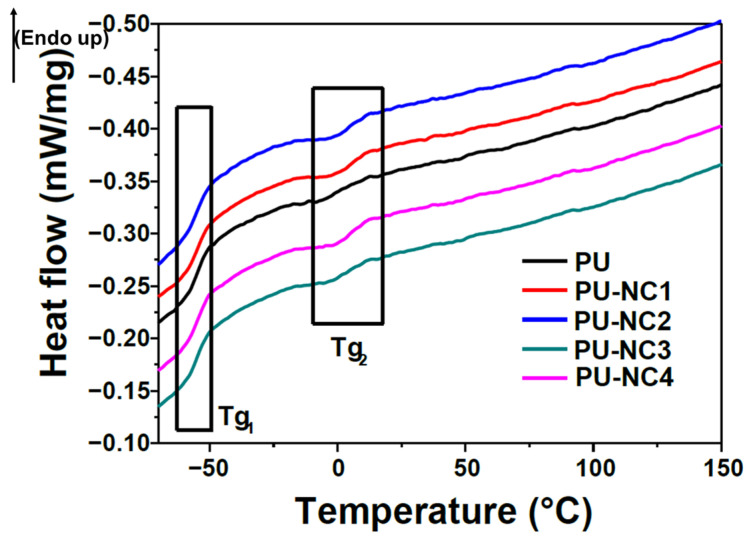
DSC curves obtained for polyurea–polyurethane–MWCNTs nanocomposites (from [80]).

Another precise method to determine the value of T_g_ consists of analysis by DSC [80] (Figure 5). Both DSC and DMA have their limitations since the T_g_ value obtained from DSC is heating rate dependent, and the T_g_ value obtained from DMA is frequency dependent. Thus, the glass transition is a kinetic process that is heavily dependent on the technique used for measurement as well as the process used to evaluate the results. Still, the thermal lag can be adjusted via calibration in both techniques mentioned [81].

DSC analysis can also provide evidence of the microphase segregation of polyurea through the presence of two step transitions on the DSC plots.

The heat generated during an explosion may rarely lead to the decomposition of the polymeric coating designed for ballistic protection applications. The properties of surface heat transfer resulting from the interaction between the shock wave and boundary layer are not completely elucidated. Local heat transfer coefficients in the collision area can increase by up to an order of magnitude, according to the literature [82]. Local heating and the development of nonuniform temperature fields on the target surface result in large temperature gradients [82]. Nevertheless, due to the short duration of contact between the heat produced by a blast and the polyurea coating, only minor localized thermal damage may happen [82]. 

**Figure 6:**
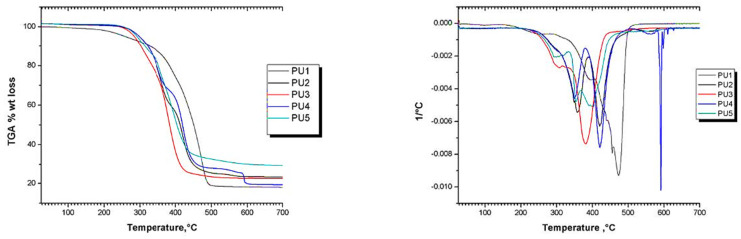
TGA analyses of polyurea samples (heating rate 10 °C/min, under nitrogen) (from [83]).

The thermal resistance of the polyurea coatings as well as the weight loss domains can be evaluated using TGA (Figure 6 and Table 4) [83].

#### 2.3.4. Impact Shielding Properties of Polyurea-Based Films Designed for Ballistic Protection Applications—Dynamic Regime Tests

**Scheme 6:**
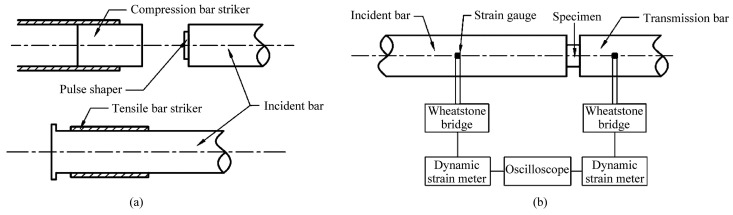
The illustration of a split Hopkinson pressure bar setup: (**a**) loading; (**b**) incident and transmission bar and measurement system [85].

**Figure 7:**
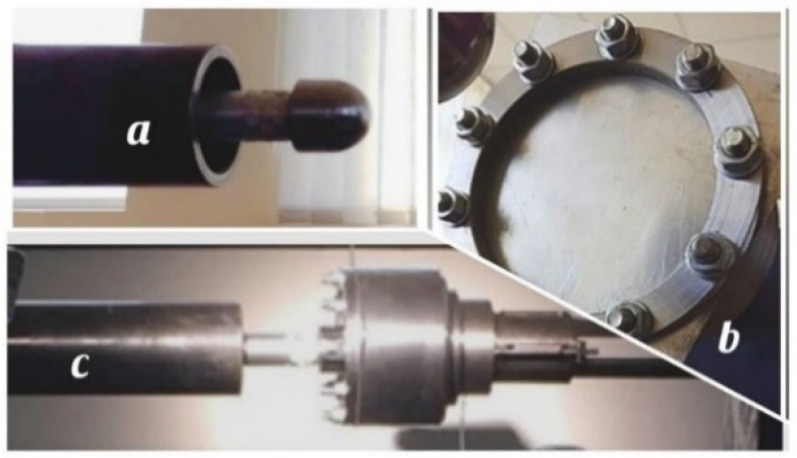
Hopkinson bar setup for experimental testing of the polyurea–polyurethane–MWCNTs-nanocomposite-coated aluminum plates in dynamic regime: (**a**) spherical head projectile; (**b**) sample before the experiment (coated on the backside with polyurea (PU) or polyurea–polyurethane–MWCNTs nanocomposite (PU-NC)); (**c**) image captured during the experiment, at the moment of the impact of the projectile on the sample (from [80]).

Fast deformation simulations, as well as impact and shock propagation models, are becoming increasingly important in design engineering [84]. Hopkinson bar experiments provide valuable insight into the material reaction when attempting to characterize the polyurea material performance under a more realistic application setting. The fundamental test setups for determining the elastic, plastic, and fracture properties of materials under high strain rates are split Hopkinson bar tests. When there are no shock waves present in the bars, simple elastic loading can produce high strains or limit loads, depending on the device. A specimen is said to be in a well-defined state of uniaxial stress. The dynamic fracture toughness of brittle materials can be determined using modifications to the standard Hopkinson bar approach [85]. An experimental setup is presented in Scheme 6 and Figure 7 [80].

**Figure 8:**
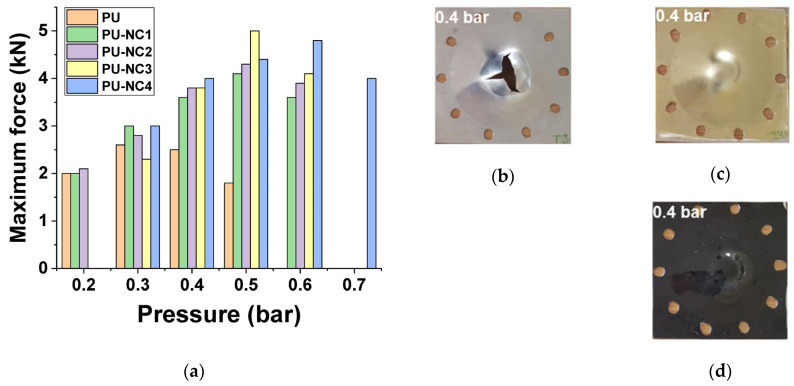
Results of Hopkinson bar experiments: (**a**) maximum force values obtained during the impact—Hopkinson bar tests; (**b**) neat metal; (**c**) polyurea-coated metal; (**d**) polyurea–MWCNT-coated metal (from [80]).

Upon impact, the materials utilized for ballistic protection suffer deformation, or they can crack or even break. Results of Hopkinson bar experiments are presented in Figure 8 and Table 5.

As can be observed from Figure 8b,c, the polyurea coating brings significant advantages in terms of impact mitigation due to its great ability to dissipate the shock wave. Thus, the polyurea coating has a critical contribution in maintaining the integrity of the metallic plate on which it is applied. Moreover, reinforcing the polyurea coating with MWCNTs (Figure 8b) led to a lower deformation of the coated metallic plate.

Based on the Hopkinson test results, an optimum filler (0.2% wt. % MWCNTs) concentration was determined for the developed polyurea formulation by Toader et al. [80]. 

## 3. Synthesis Principles for Tailoring the Final Properties of Polyurea

### 3.1. The Influence of Soft/Hard Segment Ratio on the Physical Properties of Polyurea

As expected, the ratio between the length of the soft segment compared to the length of the hard segment directly impacts the physical properties of the synthesized polymers. The phase separation at the micro scale (the segregation) represents a key factor determining the material performances [49,86]. Many outstanding publications in recent years have extensively examined polyurea microphase separation, and there is perpetual interest due to the properties of the final materials. It is widely considered that the formation of microphase-separated structures results from hard and soft segmental chemical incompatibility. If the blocks or segments are simply blended, an incompatibility may lead to a macrophase separation. When copolymerized, however, covalent bonds are formed between segments, preventing macroscopic phase separation, resulting in microphase-separated morphology. The hard segments usually self-assemble into the hard domain. However, the level of microphase separation is frequently insufficient, and some hard segments are probably dispersed as small “islands”, trapped in soft segment domains. Both the morphology and the physical characteristics of the materials are impacted by segmental intermixing within microphases [35]. In general, adding hard segments to the soft microphase can significantly increase the T_g_ of the soft microphase, which will restrict the use of the elastomers at low temperatures. Conversely, adding soft segments to hard microdomains will result in a decrease in the hard microphase temperature (T_g_) and will have an impact on the ordering behavior and crystallization of hard segments [35]. The extent of microphase separation is dictated by thermodynamic and kinetic factors. Thus, the thermodynamic factors arise from the differences between the structures of the hard and soft segments, while the kinetic factors are related to mobility of the segments and viscosity in the system. The kinetic factors depend on the: (i) polymerization method (use of prepolymers or shorter molecules); (ii) concentration of the hard segment; (iii) chemical structure and symmetry of the diisocyanate; (iv) chemical structure of the chain extenders (average chain length and polydispersity of hard segments); and (v) reaction conditions [87,88].

**Scheme 7:**
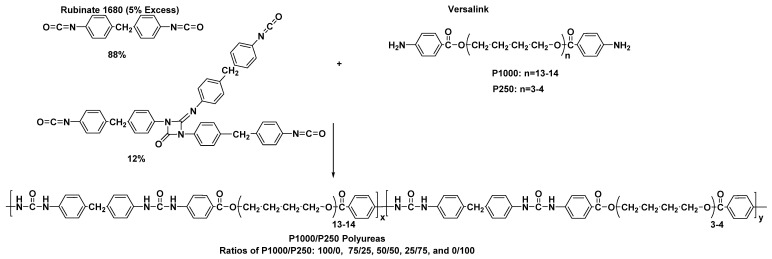
Synthesis of the two PTMO-based (M_n1_ = 1000 g/mol and M_n2_ = 250 g/mol) polyurea [86].

One of the first studies that dealt with the hard/soft segment ratio [86] presented the influence of different molecular weights of poly(tetramethylene oxide) (PTMO) on the separation on the microphase scale and on the morphology and the transition temperatures. The ratio between the two segments represented by the PTMO of two different molecular weights, M_n1_ = 1000 g/mol and M_n2_ = 250 g/mol, was systematically modified followed by bulk polymerization. The reaction components are presented in Scheme 7.

The ribbon-like glassy hard domain obtained by the self-assembly of the areas presenting the urea bond can be identified by AFM, like in the case of the soft segments based on PTMO (M_n1_ = 1000 g/mol) [86]. The overall microstructures were similar for the 100/0, 75/25, and 50/50 copolymer compositions. The hard domains became less evident as the lower-molecular-weight PTMO percentage increased. The increase in the P250 fraction up to 50% led to an increase of the mean inter-hard-domain spacing and a decrease in the extent of hard/soft segment segregation, as revealed by the SAXS (small-angle X-ray scattering) experiments. There was a further increase in P250 content for predominantly (25/75) or completely (0/100) disordered materials. Considerable changes in the dynamic T_g_ of the soft phase (or dominant mixed phase, in the case of polyurea with a high content of P250) and E′ and 25° were also registered in unlike segment mixing.

### 3.2. The Influence of the Chain Length on the Mechanical Properties of Polyurea Films

**Scheme 8:**
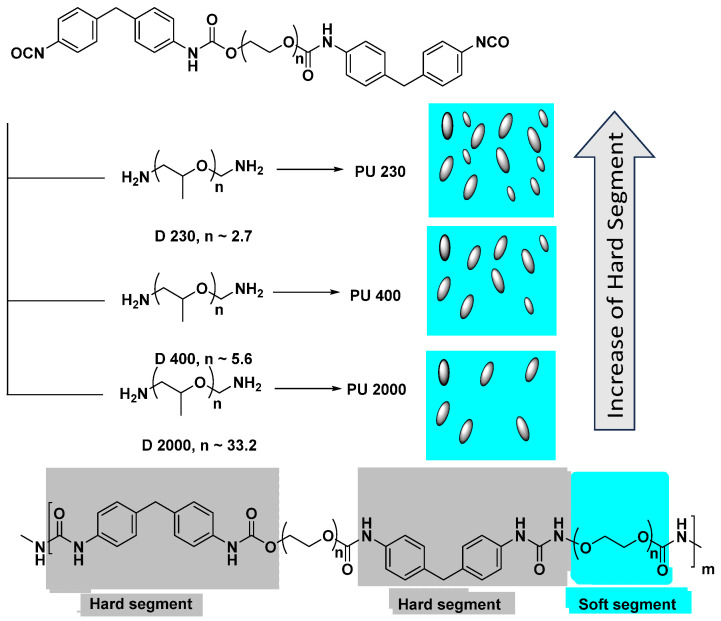
Schematic exemplification of the reaction of diisocyanate with diamines of varying molecular weight (adapted from [74]).

A more recent study [74] highlights the influence of the diamine carbon chain length on the physical properties of the final material. Scheme 8 presents the structure of the involved components in the polymerization process.

Tensile strength is inversely related to soft segment length, whereas elongation is directly proportional. The mechanical properties are the result of the phase separations at the micro level caused by the heterogeneous dispersion of the hard and soft segments. The T_g_ associated with soft segments decreased with their length increase (−43 °C for PU 2000 to −10 °C for PU 230), whereas the T_g_ associated with the hard segments remained practically constant. The DMA studies revealed that a higher frequency is required to stop the movement of chains in the soft segments. Consequently, the dynamic glass transition requires a higher frequency for the longer soft segments (10^15^ Hz for PU 2000 and 10^5^ Hz for PU 230) [74].

The morphology and the dynamic response of a series of polyurea based on MDI and PTMO (poly(tetramethylene oxide) with different molecular weights were investigated by Castagna et al. [51]. The authors determined that hard segments self-assembled into ribbon-like domains for P1000 and P650, while the degree of segregation determined by SAXS was not complete. The polyurea based on P250 presented only one phase.

Diffuse reflectance spectroscopy (DRS) (also the so-called “modulation spectroscopy” technique) provides the derivative of the spectral reflectivity (or of the imaginary part of the dielectric constant E_2_) with respect to an external parameter [89]. Thus, the dynamic process α decreases significantly when the molecular weight decreases, highlighting the mobility decrease in the soft phase that is determined by the dissolution of the hard segments. With the aid of DRS, two types of relaxation can be identified, one for the phase rich in soft segments (α) and a slow segmental (α_2_) process. The α process is present in the frequency range of the ballistic applications at ambient temperature. Thus, broadband dielectric relaxation spectroscopy was used to probe the dynamics over a broad frequency and temperature range [51]. 

Modifying polyurea by adding reactive chemical motifs, such as hydrolysable groups or dynamic covalent bonds [90], either on the polymer backbone or serving as crosslinks, is one strategy to enable the recycling of “unrecyclable” polymers [91]. Similar ideas have also been successfully applied to polyurea, which were designed to be recyclable, reprocessable, or healable by adding dynamic covalent hindered urea [92] or acylsemicarbazide [93], introducing catalysts to increase the bond dynamics, or adding auxiliary weaker bonds.

**Scheme 9:**
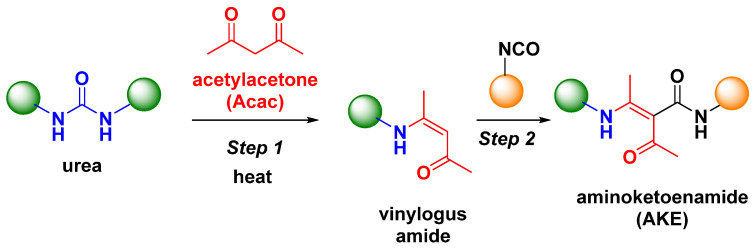
Vinylogous amide-terminated compounds for recyclable PUs (adapted from [94]).

Ma et al. [94] describe a flexible method for repurposing linear and crosslinked polyurea, which are extensively employed due to their excellent chemical stability. Di-vinylogous amide-terminated compounds (Scheme 9) can be produced in good yield by treating these polymers or their composites with acetylacetone. These compounds can react with aromatic isocyanates, and, at high temperatures, the resultant aminoketoenamide linkages are quite active.

By incorporating the Diels–Alder dynamic covalent bond, a new crosslinked polyurea with outstanding recycling and reprocessing capabilities was obtained [95]. 

### 3.3. The Influence of the Diisocyanate Structure on the Mechanical Properties of Polyurea Films

Wilkes’s group has studied the influence of the diisocyanate structure on the mechanical properties of polyurea [96,97,98]. Their studies showed that the increase in the symmetry of the diisocyanate molecule leads to a better packing of the hard domains. Thus, the increase in the diisocyanate symmetry affords enhanced mechanical properties (high modulus under ambient conditions (approx. 10^8^ Pa)), and a more rigorous segregation of the microphase takes place [96]. The “service windows” of all the polyurea were observed to be both much flatter and greater in breadth compared to their polyurethane analogs, although both contained nearly the same number of HS (approx. 13 wt. %). This can be attributed to a higher cohesiveness of the bidentate urea linkages relative to the analogous monodentate urethane linkages. The careful selection of the type and characteristics of the diisocyanate is important when attempting to anticipate/control the effect of hard segment symmetry on the structure–property relationship of polyurea and to tune the types of linkages established between the HS and SS [96]. Adhikari et al. [99] and Saralegi et al. [100] studied the influence of the symmetric isocyanate ratio on the mechanical properties and microphase separation of polyurea. They observed that a higher ordering of HS caused an increase in microphase separation in the PUs. Young’s modulus, hardness, and thermal stability all increased as a result. Tensile strength, however, appeared to be influenced by the degree of crystallinity of the HS as well as the capacity of the soft segment to generate ordered structures under strain. Joseph et al. [101] investigated the effect of altering the cis/trans-isomer distribution in poly(ether urethanes) based on 1,4-cyclohexane diisocyanate (CHDI) and discovered a rise in hardness and melting temperature of HS when the ratio of trans-CHDI was raised [101].

The 4,4′-methylenebis(phenyl isocyanate) (MDI), 4,4′-dibenzyl diisocyanate (DBDI), and equimolar combinations of these diisocyanates were employed for polyurea synthesis by Prisacariu et al. [102]. DBDI can assume a more symmetric conformation because of rotation around the ethylene bridge, which encourages the creation of crystalline hard domains, in contrast to MDI, in which the phenyl rings are linked by a methylene bridge [103]. Moreover, the dislocation of HS by stress was observed to be accompanied by a higher hysteresis. 

### 3.4. The Influence of Crosslinking on the Mechanical Properties of Polyurea Films

Crosslinking and curing are essential approaches to adjust the structure–property connection in polyurea [9]. It has been reported [9] that the physical crosslinking caused by intra- and intermolecular bidentate H bonds between the urea links is responsible for the remarkable mechanical resistance of polyurea. The deliberate addition of a chemical crosslink can modify the characteristics of polyurea even further. Adding a higher functional amine (functionality > 2) and keeping a small isocyanate excess, or a combination of both, are the most common ways to crosslink polyurea/polyurethane. Chain extenders, curing agents, or crosslinkers are aromatic or aliphatic low-molecular-weight molecules (amine- or hydroxyl-terminated with a functionality ≥ 2) that react with polyisocyanates to increase the volume of the segments with lower mobility (HS) to improve the final properties of the PU and PUR products [104]. Even though both aliphatic and aromatic chain extenders are frequently utilized, it seems that the ideal ratio of the two drives the developing macromolecule to orient in a way that promotes optimal H bonding. By adding both aromatic and aliphatic chain extenders to the formulation, the processability of polyurea using spray coating technology can be enhanced [59].

Recently, the mechanical relaxation properties of polyurea networks with various degrees of crosslinking have been studied. In terms of the crosslinking process, the hydrogen bonds specific for the hard domains determine the behavior of samples during mechanical testing. In these systems, rigid domains that are nano-segregated and connected to each other by flexible chains appear due to hydrogen bonding between urea groups. Different mechanical constraints to the chain configurations can be imposed by varying the length of the latter. The segmental motions can be restrained by chemical crosslinking, which typically induces an increase in the glass transition temperature. Moreover, crosslinking significantly increases the chemical resistance of polyurea, evidenced by the reduced swelling ratio in several organic media [9].

Two distinct segmental relaxations can be distinguished in the less constrained systems; one is linked to the embedding matrix and is faster, while the other is related to a stiffer layer that surrounds the hard domains, each of which has a different glass transition temperature. Because the soft polymer segment has less mobility when it is pulled from both extremes by the interfacial polymer layer and is well transmitted along the soft segment, these processes tend to combine into one in the sample with the shortest flexible chain, and the glass transition temperature of the softer component rises noticeably [105]. 

The dependence of polyurea properties on the soft segment length was described by Iqbal et al. [74]. They demonstrated that the length of the soft segment is directly correlated with the frequency required to initiate the dynamic “rubber-to-glass” transition process. The optimal length of the soft segments in polyurea induces an elastomeric response at the high frequencies typical of blast loadings [74]. 

### 3.5. The Influence of the Chain Extender on the Mechanical Properties of Polyurea Films

The existence of a chain extender leads to the formation of closer urea bonds favoring the establishing of bidentate hydrogen bonds. Chain extenders are compounds with a small molecule presenting a functionality equal to or greater than two which influences the mechanical properties of the synthesized material.

Due to the immiscibility between the soft and hard segments, a phase segregation takes place at the micro scale that determines, in the end, the properties [106]. These systems are comparable to the polymer mixtures, exhibiting excellent adhesion between the phases, which is due to the formation of hydrogen bonds between the chains [107]. The chain extenders induce the phase segregation processes [108]; either they interfere or complete the formation of the hard segments [109]. The bidentate hydrogen bonds between the polyurea units determine the formation of the hard domains, which present a dual function, physical crosslinking and the reinforcing of the polymeric material [110].

Tripathi et al. [60] highlighted the influence of the molecular weight of the chain extenders on the properties of the synthesized polyurea. The materials employed and their properties are presented in Table 6.

The composition of the samples prepared with the components depicted in Table 6 is presented in Table 7. The reactivity of the aromatic chain extender (DETDA) is higher than that of the aliphatic ones; thus, by controlling the ratio between aromatic and aliphatic chain extenders, enhanced mechanical properties can be obtained compared to examples in the literature. The sample PU230Ar-63 was determined to display the best mechanical properties [60]. To enhance the microphase separation of aromatic polyurea, hydrogen bonding and π–π stacking interactions between the aromatic rings can be synergistically combined [33]. Polyurea elastomers are also made by reacting a new diisocyanate with a pyrene side chain with poly(propylene oxide) diamine, as reported in a recent study [111]. The tensile strength was increased because of the strong π–π stacking between pyrene groups and the amino group anchoring effect. 

**Figure 9:**
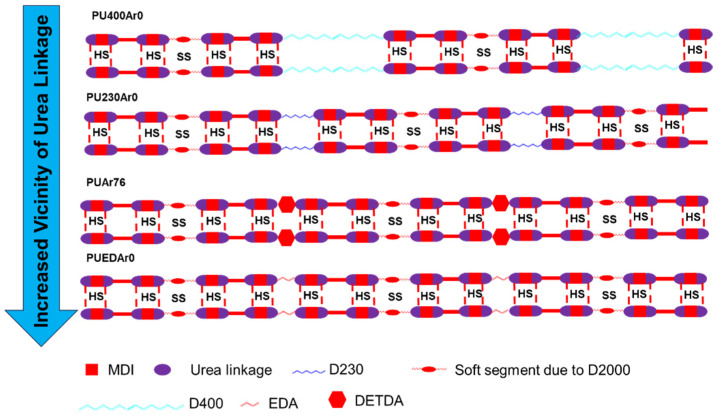
Modification of the urea linkages’ vicinity (an increase in vicinity) due to introduction of chain extender (adapted from [60]).

The optimized mechanical properties of the polyurea samples are determined by a modification of the hard/soft segment ratio and also an increase in the vicinity between the urea linkages induced by the introduction of the chain extenders (Figure 9) [60].

## 4. Self-Healing Polyureas

Supramolecular self-healing entails the use of non-covalent bonds and transient bonds, such as hydrogen bonds, π–π stacking, and metal–ligand coordination bonds, to generate the network [79,112,113]. This network can then undergo repetitive breaking and reformation, allowing multiple healing/repairing events. For dynamic covalent self-healing, covalent bonds such as disulfide bonds, Diels–Alder reactions, and imine bonds are used [114,115]. Dynamic chemical crosslinks based on exchangeable disulfide bonds were produced by reacting diamine chain extenders containing disulfide bonds with di- or triisocyanates [116,117]. The presence of disulfide bonds causes a decrease in the strength of hydrogen bonds, which can facilitate their dissociation and, in turn, promote topological network rearrangement. Furthermore, the use of aromatic disulfides can allow modification of the hard-to-soft-segments ratio, leading to enhanced mechanical properties and self-healing characteristics [116].

The reversibility of the physical crosslinking can be enhanced by using chemical agents that lead to the formation of chemical bonds that assure a higher mechanical and chemical resistance. The implementation of a chemical crosslinking determines the impossibility of the material recycling, which represents an important drawback; therefore, a reversible chemical crosslinking (debonding and re-bonding, depending on the external conditions) [90] would bring multiple benefits. Thus, the incorporation of aromatic disulfides in the hard segments and as chain extenders could offer a solution for the recyclability characteristics [90,118,119].

**Figure 10:**
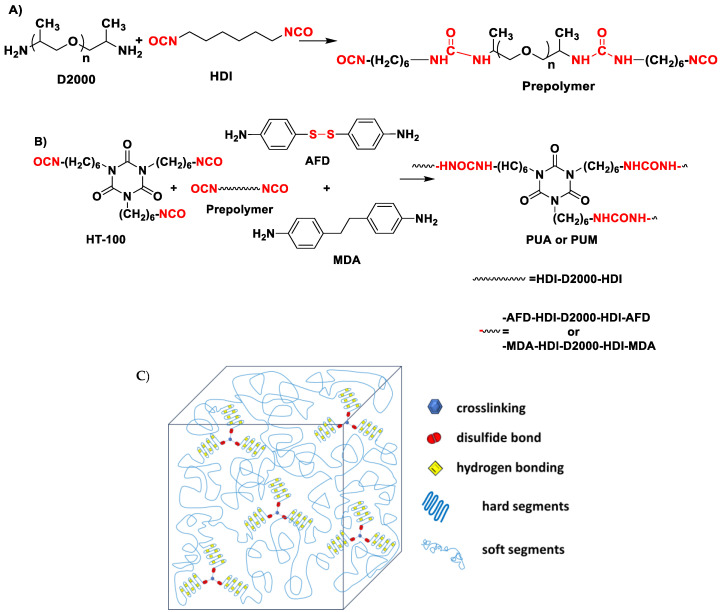
(**A**) Synthesis of the prepolymer. (**B**) Synthesis of the polyurea (PUA) containing disulfide bonds and control samples (PUM). (**C**) Schematic representation of dynamic crosslinking (adapted from [117]).

Li et al. [117] presented the utilization of a diisocyanate with disulfide bonds used as chemical crosslinker. The synthesis is presented in Figure 10.

**Figure 11:**
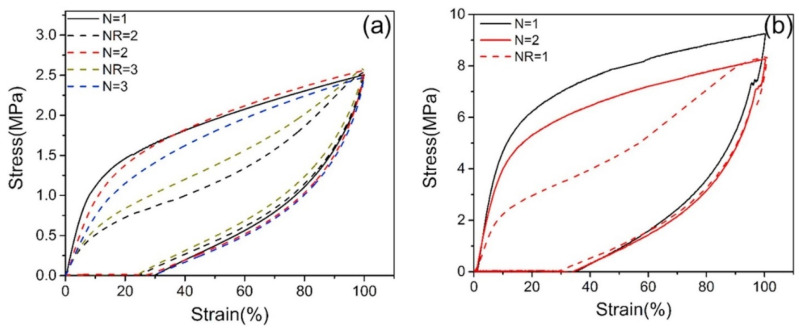
Cyclic stress–strain curves of PUA (**a**) and PUM (**b**). N = 1 represents the first tensile cycle without any treatment. N = 2 or 3 means the second or third tensile cycle of PUA and PUM after heating at 150 °C for an hour. NR = 2 and NR = 3 represent the second or third tensile cycle after recovery at room temperature for 1 h and 24 h, respectively (adapted from [117]).

To demonstrate the rearrangement of this dynamic network, the dynamic hysteresis (cyclic stress–strain testing with or without treatment) was registered (Figure 11). When comparing the first load–unloading cycle test with the second cycle test, which appears unmodified but it also involves the exchange reaction, network reformation can be observed. In contrast with the PUM control sample that had an incomplete recovery, the loss registered being 14%, the PUA demonstrated the ease with which the bond can be reformed in a dynamic regime. 

**Scheme 10:**
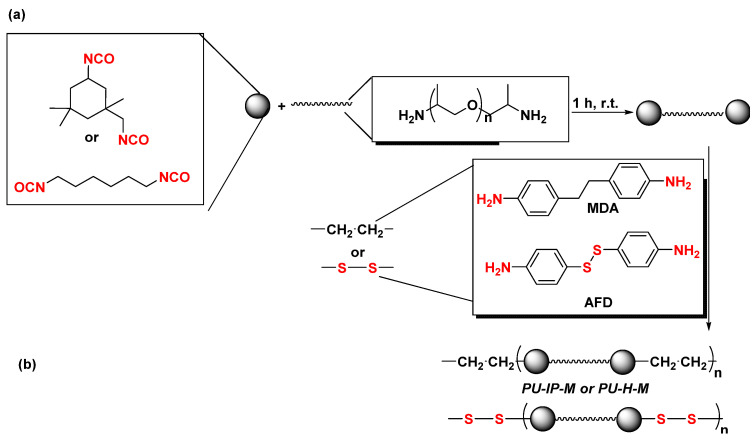
Synthesis route for the four polyurea samples (adapted from [119]): (**a**) cyclic or linear aliphatic diisocyanate and chain extenders examples; (**b**) polyurea structure with or without dynamic bonds.

A more recent study [119] presents the influence of type of diisocyanate employed and the chain extender influence on the self-healing capacity of the polyurea. The synthesis route for the four polyurea samples is presented in Scheme 10.

Li et al. [119] determined that the structure of the diisocyanate has a greater influence on the self-healing capacity compared to the disulfide bond. The combination between steric hindrance due to the isocyanate groups and the existence of disulfide bonds favors the packing of the hard segments and the self-healing capacity.

## 5. Polyurea-Based Nanocomposites

The introduction of multiwall carbon nanotubes (MWCNTs) in the polyurea matrix leads to an increase in the glass transition temperature (T_g_) in addition to the enhancement of the mechanical properties [8]. Materials with mechanical properties that are better than epoxy resin can be created by using this reinforcing technique [120].

### 5.1. Polyurea Nanocomposites Based on Functionalized Nanofillers

Carbon nanotubes can be employed pristinely or after prior functionalization. In a relatively recent study [121], the influence of unfunctionalized nanotubes on polyurea properties was studied. The nanocomposites exhibited a tensile stress approximately 100 times higher than that of polyurea. The enhancement of the mechanical properties can be explained by the increase in the crosslinking density determined by the MWCNTs, which facilitates the formation of hydrogen bonds and secures/locks/immobilizes the polymer chains.

**Scheme 11:**
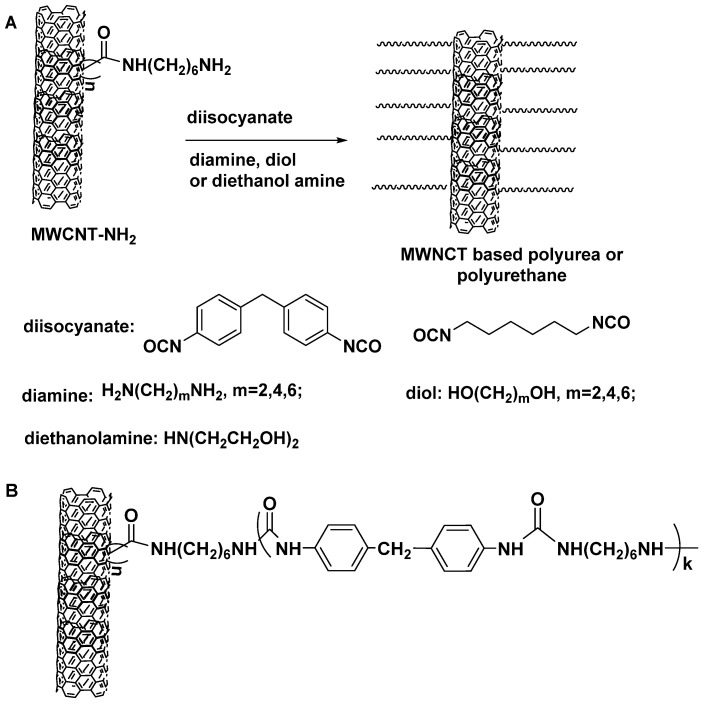
Schematic illustration of the MWCNT functionalization by the in situ polycondensation approach (**A**) and the chemical structure of the MWCNT–polyurea obtained using MDI and 1,6 hexamethylene diamine (**B**) (adapted from [122]).

Gao et al. [122] used MWCNTs functionalized with amino groups, as presented in Scheme 11.

Using the approach presented in Scheme 11, Gao et al. [122] observed the formation of a core–shell morphology, demonstrating by TEM analysis the twisting of the polymer matrix around the MWCNTs. The MWCNTs’ Raman characteristic signals were strongly attenuated in the presence of the polymer, which confirms their functionalization, while the SEM analysis revealed the formation of arc, flat, rose flower-like structures, which constitutes a new insight into supramolecular chemistry assemblies [122].

**Scheme 12:**
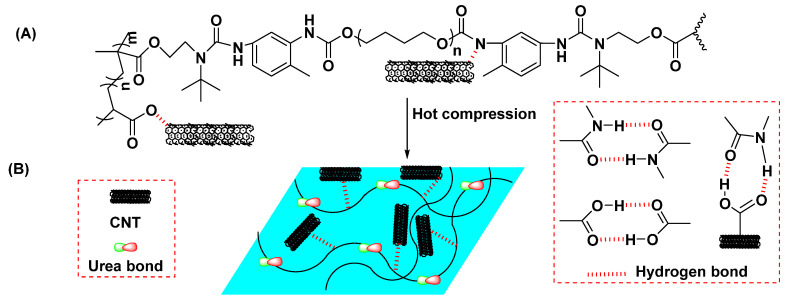
Schematic representation of the fabrication steps for crosslinked polyurea—(**A**) obtaining of crosslinked polyurea by photo-induced polymerization using acrylic acid, 2-(tert-butylamino)ethyl methacrylate, toluene 2,4-diisocyanate, and polytetramethylene ether glycol (M_n_ = 1000 g/mol); (**B**) schematic illustration of the hydrogen bonds inside the nanocomposite matrix (adapted from [123]).

A more recent study [123] presents the synthesis and characterization of a nanocomposite with carbon nanotubes as reinforcing agent which displays self-healing capacity, making it an ideal candidate for recycling processes (Scheme 12). 

**Scheme 13:**
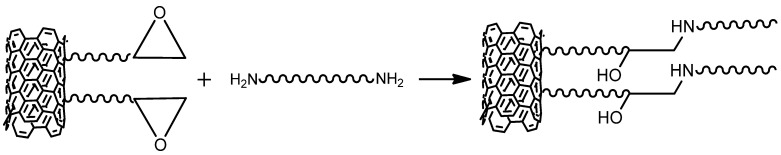
Reaction between the epoxy functionalized MWCNTs and PPG (adapted from [120]).

Zhou et al. [123] obtained a polyurea–MWCNT composite with self-healing characteristics and memory shape properties (Scheme 13) by photo-induced polymerization and hot molding to enhance the compatibility between the functionalized carbon nanotubes and the polymer matrix [123]. The obtained nanocomposites displayed excellent thermal properties, including self-healing after near-infrared irradiation. The authors established that the optimum nanofiller concentration is 1%. 

Another type of functionalization of the carbon nanotubes employed in polyurea composites involved the introduction of oxirane groups by grafting poly(glycidyl methacrylate) to the MWCNTs’ surface [120], which afforded an efficient dispersion in the polyurea matrix in conjunction with poly(propylene glycol) bis(2-aminopropyl ether) (PPG) (M_n_ = 2000 g/mol) and 4,4′ -diamino diphenylmethane (Scheme 11).

The tensile tests confirmed an important mechanical properties enhancement up to a filler concentration of 0.2% (weight %), after which a decrease was observed. These nanocomposites presented a high thermal stability of over 300 °C [120].

**Scheme 14:**
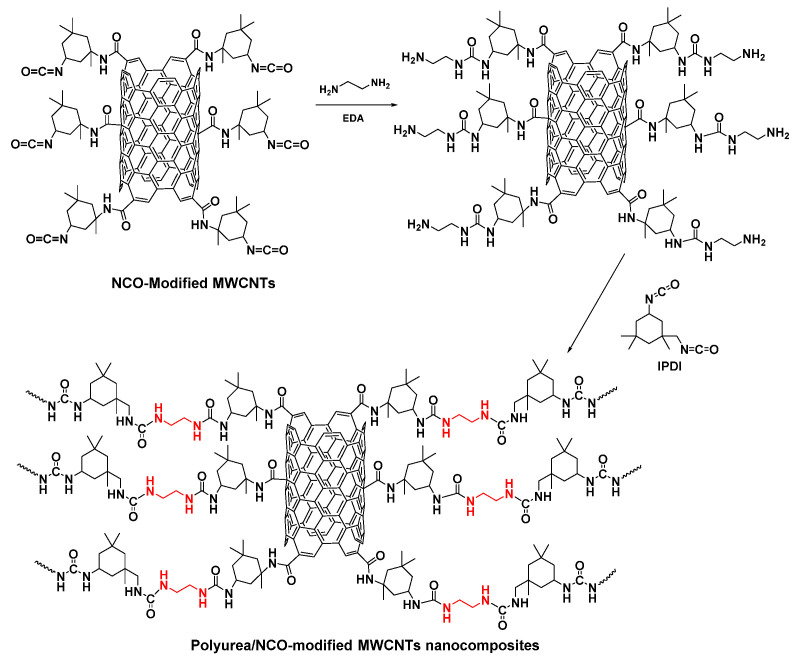
Reaction between NCO-modified MWCNTs, EDA, and IPDI (adapted from [124]).

Jagtab et al. [124] presented the synthesis of microcapsules with the tunable mechanical properties necessary for applications that are sensitive to pressure. MWCNTs are incorporated in the walls of the microcapsules by polymerization at the interface. The specific reactions are presented in Scheme 14.

Layered silicates are another type of compound used to reinforce polyurea, to increase the Young’s modulus and the elongation. Cai and Song [125] demonstrated that the macromolecular structure is crucial to reinforcing the polyurea. Thus, the addition of C20 organoclay has a more significant impact on the mechanical properties as the crosslinking density is higher.

The excellent resistance to impact and the high thermal resistance are properties that can be optimized through the use of polyhedral oligomeric silsesquioxane nanoparticles (POSS) [126]. Also, the polyurea/eutectic composites (those exhibiting negative Poisson ratio values) can enhance the blast resistance of the specific formulations [8].

The studies were focused on comparative analyses between the different influences of exfoliated graphene nanoparticles and POSS on the polyurea matrix. Thus, impact traces were investigated by 3D tomography analysis [127]. The analyses showed that the polyurea with POSS have a larger number of voids, but the voids have smaller dimensions. Through computational techniques, the authors showed that the stress is not uniformly distributed; the voids have an aleatory size and shape.

The existence of the nanoparticles in the polyurea composition affects the segmental mobility [128,129], which is reflected in the values of the T_g_, and subsequently in the response to the blast impact. The comparative influence of MWCNTs, POSS, and nanoclay was investigated by Roland et al. [130]. The authors calculated the amount of energy dissipated by the materials using the peak areas from the DMA analysis. The glass transition temperature and loss modulus curves obtained by Roland et al. [130] are presented in Table 8.

The glass transition temperature was not influenced by the introduction of nanoclay, while MWCNTs determined a similar modification at half the concentration [130]. The penetration velocity did not vary significantly, except in the case of POSS, which exhibited an increase explainable by the active participation in the polycondensation reaction [130].

In the case of supersonic projectiles, Dewapriya and Miller presented simulated ballistics tests of multilayer polyurea/silicon carbide (SiC) nanostructures using molecular dynamics [131]. Dewapriya et al. [131] demonstrated through a mathematical molecular dynamics model that the ballistic limit velocity and the specific penetration energy of the polyurea/SiC multilayers are significantly higher than the experimentally measured values of other protective materials.

### 5.2. Self-Healing Nanocomposite Polyurea

**Figure 12:**
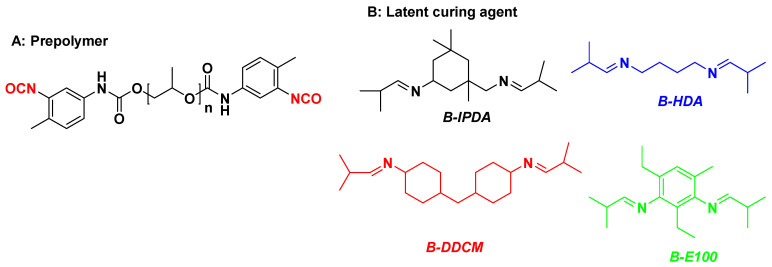
Components of the one-component polyurea with blocked amines sensible to humidity (adapted from [115]).

Impact-resistant materials are resilient to impact due to their high modulus or ability to absorb energy (cushion role) [132,133,134,135]. Based on this behavior, they can be categorized into two groups: soft materials, such as foams and gels, and hard materials, such as metals, ceramics, and composites. Taking all these into account, it is possible to create self-healing polyurea-based protection materials through a proper polymer network design [136]. Considering that the reaction between the diisocyanate and the polyamine is quite fast, the application requires special attention or the use of a solvent to decrease the reaction rate. As a result, controlling the rate of polymerization is a challenge that can be met by using a secondary amine; however, this reduces the number of hydrogen bonds, which impacts the mechanical properties. Another way to control the reaction rate is to use Schiff bases that can block the amine in the absence of humidity (Figure 12) to allow its release and also facilitate the self-healing process in the presence of water [115]. Thus, the polyurea materials obtained by using the diamines blocked by Schiff bases present the advantage of self-healing capacity and good impact force attenuation coupled with good tensile resistance [115]. 

**Figure 13:**
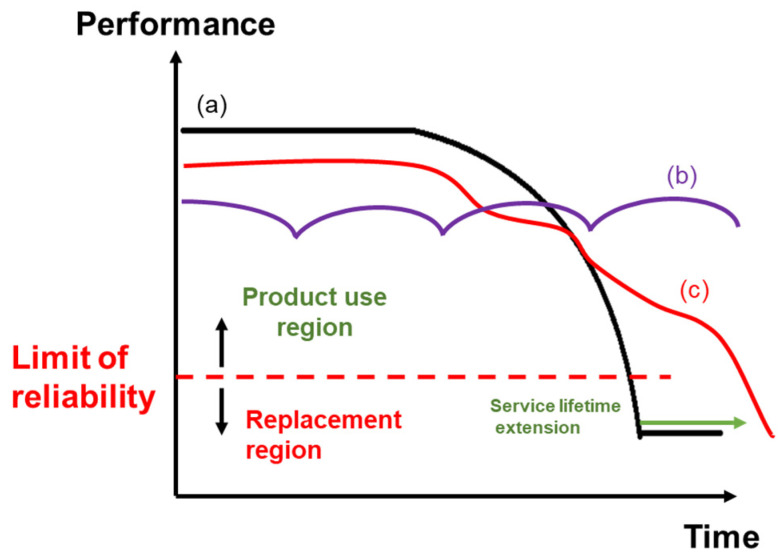
Performance plotted against time for (a) normal material, (b) ideal self-healing material, and (c) self-healing material (adapted from [79,137]).

In Figure 13, the self-healing process is quantified as the variation of the materials’ performance with time [79,137]. Thus, the material repairing/regeneration process can be achieved by the incorporation of the healing agents in microcapsules embedded in the polymer matrix that release their content upon breaking and restore the chemical bonding [137]. In terms of the mechanism, the self-healing process can also involve intermolecular diffusion [138,139], non-covalent supramolecular interaction [140], or dynamic covalent bonds [42].

**Scheme 15:**
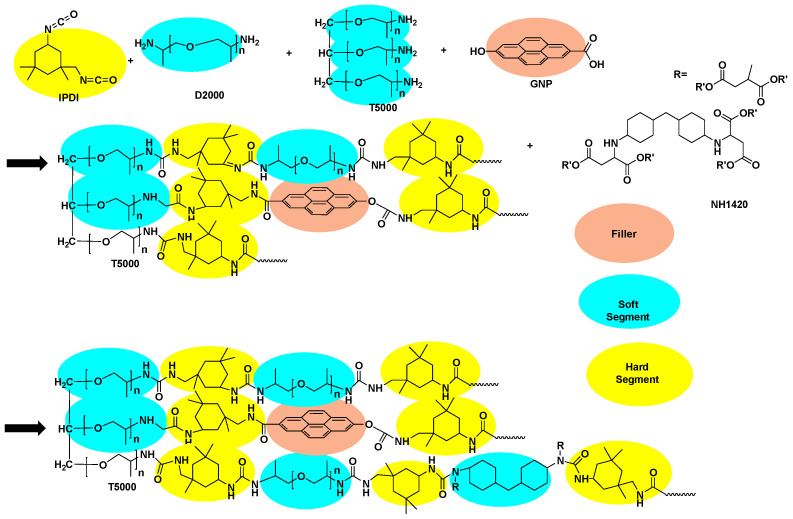
Polyaspartic polyurea-functionalized graphene nanocomposites (polyurea/IP-GNP) (adapted from [141]).

A very intriguing family of materials with a T_g_ value significantly lower than room temperature is polyurethane elastomers. These materials have appealing mechanical and thermal qualities that make them useful for a variety of applications. In materials science and polymer composites, graphenes became the “superstar” nanofiller, demanding particular attention for both chemical and non-chemical functionalization. The covalent or non-covalent functionalization of the graphenes is necessary to facilitate the compatibilization between the carbon-based nanofiller and the polymer matrix. The covalent functionalization is more efficient and can ensure that the nanofiller has a homogeneous distribution in the polymer matrix. Using a polyisocyanate modified with graphenes, Meng et al. [141] demonstrated the synthesis of polyaspartic polyurea elastomers (Scheme 15). In comparison to pure polyurea, the presence of functionalized graphenes of 0.05 vol% of IP-GNPs resulted in a tensile strength increase of 108.2% and outstanding resistance to acidic and alkali corrosion. Thanks to the hydrogen bonding, the final graphene-based polymer nanocomposite exhibited an 80% healing efficiency after 9 h of treatment at 60 °C. 

### 5.3. Hybrid Polyurea—Polyurethane Matrices from Renewable Sources

**Scheme 16:**
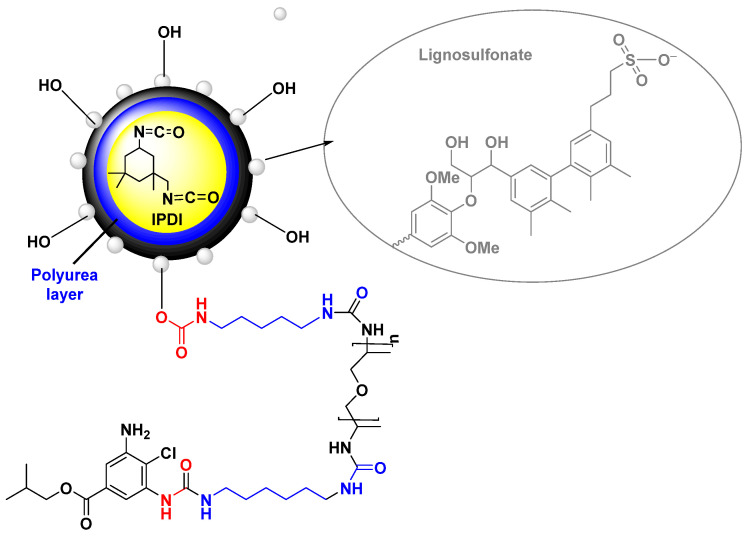
Synthesis strategy of the self-healable polyurea based on lignin sulfonate microcapsules (adapted from [142]).

A current trend in materials science is to introduce renewable components into the formulation. Qian et al. [142] presented an example of the utilization of encapsulated lignin to achieve self-healing characteristics. By this application, it was demonstrated that the lignin capsules are stable to UV light, which prolongs the reactivity and therefore increases the lifetime of the polyurea self-healing coatings. The microcapsules are obtained by reacting a lignin sulfonate presenting a high number of hydroxyl groups with the isocyanate groups of the prepolymer to generate the shell (Scheme 16).

**Scheme 17:**
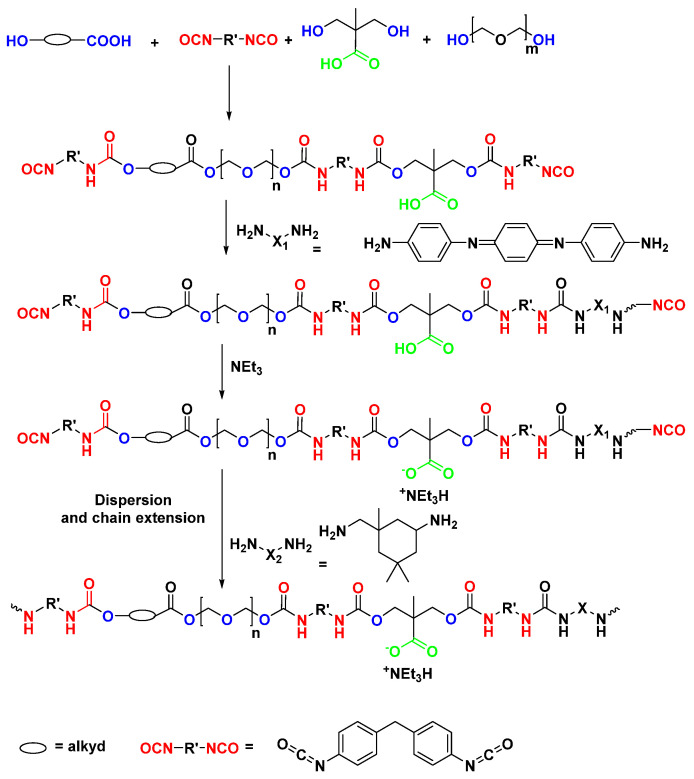
Synthesis of biobased air-drying water-borne polyurea dispersions (from [143]).

In materials engineering, the synthesis of hybrid polyurea for biobased formulations is another current research focus. An example of several studies related to water-borne polyurea modified by aniline trimer was developed by Zeng et al. [143]. A hydroxyl-rich alkyd intermediate based on biobased linoleic acid along with polyether polyol N-210, 4, 4′-dicyclohexylmethane diisocyanate, 2,2-Bis (hydroxymethyl) propionic acid, isophorone diamine, and an aniline trimer (as a partial chain extender) was used to prepare biobased air-drying water-borne polyurea dispersions (Scheme 17). The studies were focused on the correlation between the self-healing performance of the biobased air-drying water-borne polyurea dispersions and paint films and the content of alkyd intermediate and aniline trimer [143].

Diisocyanates production is continuously increasing considering the market growth of polyurethanes and polyurea, while the necessities of environment protection and human health have driven research studies to the synthesis of biobased isocyanates for biobased polyurea formulations. As an example, Vencorex Chemicals (Saint-Priest, France) released into the market an aliphatic diisocyanate called Tolonate™ X FLO 100 derived from palm oil [144]. In Table 9, a list of the most interesting structures for the biobased isocyanates is presented in accordance with the literature data [144].

**Scheme 18:**
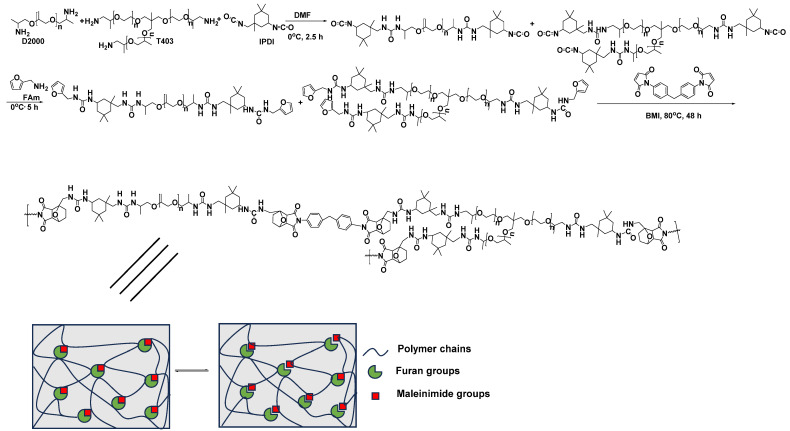
Synthetic procedure of polyurea formulation based on dynamic covalent bonds (adapted from [95]).

Nevertheless, the recycling and reprocessing of polyurea remain important challenges, especially at temperatures below 100 °C. An innovative method for improving the reuse/recycling of polyurea-based materials was proposed by Wei et al. [95] by introducing polyurea Diels–Alder capable groups into the structure for the implementation of dynamic covalent bonds. As a result, the tensile strength and elongation at the break of crosslinked polyurea could reach up to 15.24 MPa and 529.2%, respectively, keeping the mechanical properties almost unchanged even after repeated processing. As expected, (Scheme 18), the synthesis route highlighted the concept of the reactions that involve cyclic destruction and regeneration steps of a 3D structure. 

**Figure 14:**
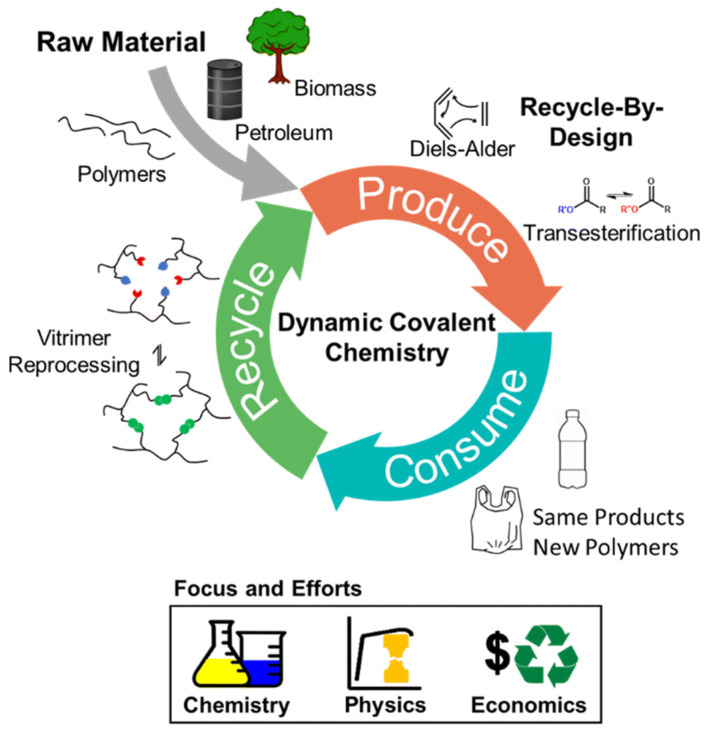
Closed loop from production to recycling achieved by material design—the introduction of dynamic covalent bonds (based on, for example, Diels–Alder cycloadditions or transesterification) facilitates the recycling process, while the introduction of biobased components reduces the stress on non-renewable raw materials (from [148]).

Thus, the use of dynamic covalent bonds (based on Diels–Alder cycloadditions or transesterification) introduces new possibilities for polyurea recycling processes on the one hand, while the introduction of biobased components reduces the stress on non-renewable raw materials on the other (Figure 14). 

## 6. Conclusions and Perspectives

Polyureas constitute one of the most important classes of polymers with applications in different domains such as coatings, adhesives, sealants, and insulation materials. In general, for many applications, polyurea is preferable to polyurethanes due to a high reaction rate and some physical properties such as resistance to chemical agents and thermal resistance. Regarding ballistic applications, there are two large classes of compounds used: the very rigid ones and the flexible ones. The advantage of the flexible ones is that, after the impact, they do not respond as violently as the rigid ones. Polyureas are an extraordinary case because they contain both rigid segments, which are due to the diisocyanates used and to the hydrogen points formed, and a flexible zone, which is due to the chemical structures of the polyamines.

The problems that arise during the usage of polyurea consist primarily of the difficulty in restoring a defect, which can appear on impact, and controlling the reaction rate during deposition. This aspect was, to a small extent, tackled through the development of self-healing films, but the mechanical properties are not so important. Thus, the first future development direction would go towards this area, and the second towards the fully biobased ones, because we are at a stage where fossil resources are limited, greatly reduced, and sometimes not environmentally friendly. For this reason, it seems extremely attractive to create polyurea that are biobased and also have remarkable mechanical properties. Due to the current conflicts, the use of polyurea from a ballistic point of view is a must. Thus, the improvement of polyurea-based materials for blast protection will receive much attention with the development of novel composites with self-healing and reversible crosslinking capacity. The second research direction that will certainly receive an increasing amount of attention is the integration of renewable components to reduce the environmental impact of the materials. Considering the current geopolitical situation, the ballistic application must receive continuous attention. Nonetheless, environmental considerations must also be considered when synthesizing materials for the future; the use of renewable, non-toxic (isocyanate free) raw materials and the facilitation of recycling and or repurposing must be at the core of the material design.

## Figures and Tables

**Table 1:** Performance characteristics of typical polyurea coatings.

Parameter	Value Range
Tensile strength, MPa	7.5–27.5
Shore hardness	A20–D65
Elongation, %	20–1000
Modulus, MPa	3.4–13.8
Water absorption, %	5–16
Abrasion resistance, mg weight loss	6–70
Impact resistance, in kg	25–90

**Table 2:** Tensile properties of poly(ether urea) formed by stoichiometric reaction of amine terminated poly(tetramethylene oxide) (M_n_ = 1000 g/mol) and different types of diisocyanates (adapted from [40]).

Isocyanate Type	Hard Segment Content (wt. %)	Tensile Strength (MPa)	Ultimate Elongation (%)
Isophorone diisocyanate (IPDI)	16.8	7.15	1070
Bis(4-isocyanatocyclohexyl)methane (HMDI)	19.3	19.6	925
2,6- and 2,4-toluene diisocyanate (TDI)	13.7	12.3	760
p-diphenylmethane diisocyanate (MDI)	18.5	16.9	670
1,3-phenylene diisocyanate (MPDI)	12.7	5.4	700
1,4-phenylene diisocyanate (PPDI)	12.7	19.5	540
1,6-hexamethylene diisocyanate (HDI)	13.2	24.7	760
1,4-cyclohexyl diisocyanate (CHDI)	13.1	30.0	980

**Table 3:** Polyurea brands marketed for ballistic protection and their mechanical properties [2].

No.	Brand	Year	Mechanical Properties Details				Reference
			Tensile Strength (MPa)	Elongation (%)	Tear Strength (kN/m)	Shore Hardness	
1	EP JS	2008	20.34	350	87.5		[61]
2	Dragon Shield-BC	2014	11.75	50	-	-	[62]
3	HM-VK	2016	10	500	-	-	[63]
4	Link-XS350	2019	22.39	163	-	60 ± 1 HD	[64,65]
5	SWD562	2019	16.5	160	-	-	[66]
6	SPUA 306	2019	24	400	85	85–95 HA	[14]
7	SPUA 307	2019	25	45	81	65–75 HD	[14]
8	AMMT-53	2020	25	50	90	70 HD	[28,67]
9	AMMT-55	2020	35	300	115	65 HD	[28,67]
10	AP103	2021	16	450	-	-	[68]
11	-	2021	18	350	45	90–96 HA	[22]
12	Qtech T26	2022	25.4	451.88	75.5	-	[69]

**Table 4:** The initial decomposition temperature (T_initial_), 50% weight loss temperature (T_50%_), and final decomposition temperature (T_end_) of polyurea samples (from [83]).

Sample	Decomposition Temperature, °C		
	T_initial_	T_50%_	T_end_
PU-1	141	452	517
PU-2	155	387	642
PU-3	176	374	446
PU-4	164	409	700
PU-5	177	390	700

**Table 5:** Maximum force values obtained during the impact tests with Hopkinson bar (from [80]), PU-NC2—0.1% MWCNTs; PU-NC3—0.2% MWCNTs; PU-NC4—0.3 %MWCNTs (weight %).

Pressure (Bar)	Aluminum Plate (Uncoated)	Aluminum Plate (Coated with PU)	Aluminum Plate (Coated with PU-NC2)	Aluminum Plate (Coated with PU-NC3)	Aluminum Plate (Coated with PU-NC4)
	Maximum Force (kN)	Maximum Force (kN)	Maximum Force (kN)	Maximum Force (kN)	Maximum Force (kN)
0.2	2.0	2.0	2.1	-	-
0.3	2.6	3.0	2.8	2.3	3.0
0.4	2.5	3.6	3.8	3.8	4.0
0.5	1.8	4.1	4.3	5.0	4.4
0.6	-	3.6	3.9	4.1	4.8
0.7	-	-	-	-	4.0

**Table 6:** Characteristics of components used in polyurea formulations (adapted from [60]).

Component	Role	Equivalent Number *n*_eq_	Viscosity η_30 °C_ (MPa s^−1^)	Density ρ (g cm^−3^)	Structure
SUPRASEC 2054	Major monomer	3.6	880	1.09	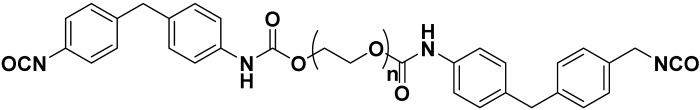
Propylene carbonate	Diluent	—	2	1.20	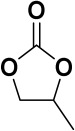
Ethylene diamine	Chain extender	33.3	1.5 × 10^−3^	0.90	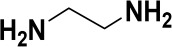
JEFFAMINE D-230	Chain extender	8.1–8.7	7	0.93	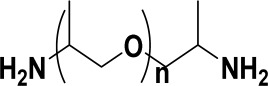 n ≈ 2.5
JEFFAMINE D-400	Chain extender	4.1–4.7	21	0.96	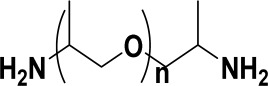 n ≈ 6.1
JEFFAMINE D-2000	Major monomer	0.9–1.0	182	0.98	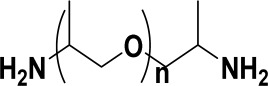 n ≈ 33
DETDA	Chain extender	11.2	106	1.02	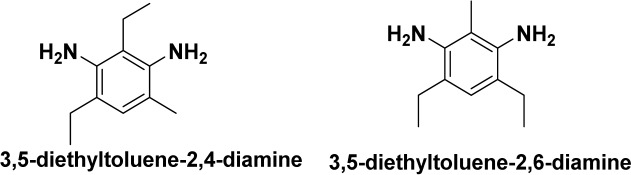

**Table 7:** Formulations for the polyurea prepared using the components from Table 6 (adapted from [60]).

Sample Code	Amine (Part per Unit Volume)					HS (%)	Contribution of Aromatic Chain Extender (%)
	Chain Extender				D-2000		
	EDA	D-230	D-400	DEDTA			
PUAr-76	—	—	—	21.22	78.78	60.86	76
PU230Ar-63	—	5.48	—	17.52	77.00	61.65	63
PU230Ar-36	—	16.41	—	11.05	72.54	63.72	36
PU230Ar-10	—	27.35	—	2.68	69.97	64.84	10
PU230Ar-0	—	31.30	—	—	68.70	65.43	—
PU400Ar-62	—	—	10.94	17.25	71.81	64.25	62
PU400Ar-47	—	—	21.88	13.28	64.84	66.65	47
PU400Ar-33	—	—	32.82	9.3	57.88	71.06	33
PU400Ar-19	—	—	43.76	5.53	50.91	74.49	19
PU400Ar-0	—	—	58.42	—	41.58	79.1	—
PUEDAr-32 ^a^	5.65	—	—	9.85	84.50	61.74	32
PUEDAr-0 ^a^	8.65	—	—	—	91.35	54	—

^a^ Prepared using a batch process.

**Table 8:** Glass transition temperatures for polyurea composites with MWCNTs, POSS, and nanoclay and penetration velocity (V-50) (from [130]).

Coating	T_g_ (°C)	Peak *G*″ (Mpa)	V-50 ^a^
Neat	−68.7	62	1.3
5% Nanoclay	−68.7	67	1.29
2% POSS	−63.9	76	1.34
1% MWCNT	−63.2	62	1.30

Note: ^a^ Relative to 7 mm RHA.

**Table 9:** Biobased isocyanates for biobased polyurea formulations envisaged by Niesiobędzka and Datta [144].

	NCO%	Biomass (%)	Viscosity (mPa·s/25 °C)	Company	Ref.
Tolonate™ X FLO 100	13 ± 1	25	140 ± 80	Vencorex	[145]
	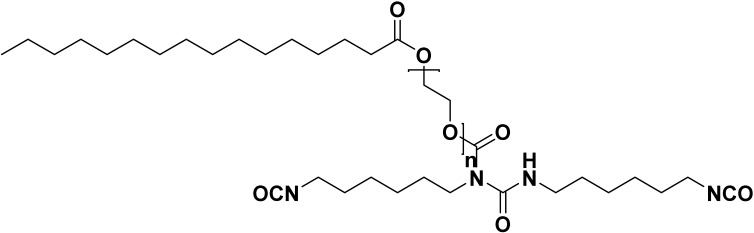				
Desmodur^®^ eco N 7300	21.5	70	9200	Covestro	[146]
	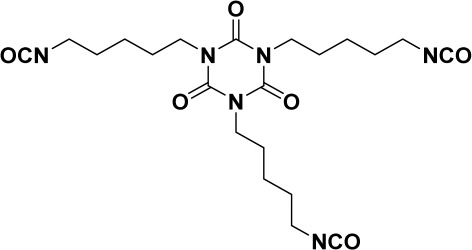				
STABIO™ D-370N	25	70	2.0	Mitsui Chemicals	[147]